# Monitoring Wheat Lodging at Various Growth Stages

**DOI:** 10.3390/s22186967

**Published:** 2022-09-14

**Authors:** Shuangshuai Jiang, Jinyu Hao, Han Li, Changzhen Zuo, Xia Geng, Xiaoyong Sun

**Affiliations:** 1 Agricultural Big-Data Research Center, College of Information Science and Engineering, Shandong Agricultural University, Tai’an 271018, China; shuangshuai1999@163.com (S.J.); jinyuhao96@126.com (J.H.); lhan98@126.com (H.L.); zuo109528@163.com (C.Z.); 2 Department of Data Science, College of Information Science and Engineering, Shandong Agricultural University, Tai’an 271018, China

**Keywords:** unmanned aerial vehicle (UAV), wheat lodging, deep learning, growth stages, area calculation

## Abstract

Lodging is one of the primary factors that reduce wheat yield; therefore, rapid and accurate monitoring of wheat lodging helps to provide data support for crop loss and damage response and the subsequent settlement of agricultural insurance claims. In this study, we aimed to address two problems: (1) calculating the wheat lodging area. Through comparative experiments, the SegFormer-B1 model can achieve a better segmentation effect of wheat lodging plots with a higher prediction rate and a stronger generalization ability. This model has an accuracy of 96.56%, which realizes the accurate extraction of wheat lodging plots and the relatively precise calculation of the wheat lodging area. (2) Analyzing wheat lodging areas from various growth stages. The model established, based on the mixed-stage dataset, generally outperforms those set up based on the single-stage datasets in terms of the segmentation effect. The SegFormer-B1 model established based on the mixed-stage dataset, with its mIoU reaching 89.64%, was applicable to wheat lodging monitoring throughout the whole growth cycle of wheat.

## 1. Introduction

Since wheat is one of the most widely planted crops in the world [1], its stable production is of great significance to national food security. Lodging is one of the primary factors that reduce wheat yield [2]. Typhoons, heavy precipitation, plant diseases and pests, excessive application of nitrogen fertilizers, etc., may tilt or break wheat roots, resulting in lodging [3]. Severe lodging can reduce wheat yield by 27% [4]. Accurate monitoring of wheat-lodging plots helps provide data support for post-disaster emergency responses and the subsequent settlement of agricultural insurance claims [5]. Therefore, it is particularly important to rapidly and accurately investigate and monitor wheat-lodging plots after a disaster occurs [6].

Currently, wheat lodging is mainly manually monitored. Manual monitoring not only has many shortcomings, e.g., long time span, high labor cost, low efficiency and large measurement errors [7,8], but may also cause secondary damage to the wheat during field evaluation [9]. Satellite remote sensing technology has been increasingly applied to monitor crop lodging [10]. In terms of data acquisition speed, unmanned aerial vehicles (UAVs) have better real-time performance than satellites [6], while satellite data is greatly affected by the weather or climate with a long access period [11]. Compared with traditional remote sensing platforms, UAVs can provide strong data support for crop phenotype research with a sufficient spatiotemporal resolution for their advantages, such as low operating cost, simple operation, high spatial resolution, ability to fly in cloudy conditions and strong capability for real-time monitoring [12,13,14]. UAV remote sensing monitoring has been widely used in the field of crop monitoring [15,16,17].

With the constant development of deep learning in the field of computer vision, deep-learning technology has been increasingly and extensively used to study crop phenotypic information [18,19,20]. Unlike traditional algorithms, deep learning manages to automatically extract effective features through a multi-layer neural network [21,22]. In particular, the neural network model can extract the feature images of local details, as well as the senior semantic features of images. The semantic feature is the feature information obtained after several feature extractions. One study has shown that the deep learning algorithm has a stronger potential than traditional learning algorithms for application in complex scenarios when it comes to the extraction of crop lodging information based on deep learning [3]. Many studies have been carried out on the monitoring of wheat lodging, and many convolutional neural network (CNN)-based methods have been successfully applied to lodging identification [23]. Zhao et al. [9] proposed a rice lodging evaluation method based on the UNet. A UAV equipped with a high-resolution digital camera and multi-spectral camera was used to collect the images of lodging and non-lodging rice, simultaneously. The dice coefficients of red, green and blue (RGB) images and multispectral image test sets were 0.9442 and 0.9284, respectively, and the effect of identifying rice lodging from RGB images without feature extraction was better than that from multispectral images. Mardanisamani et al. [24] put forward a deep convolution neural network (DCNN) for lodging identification. Five spectral channel forward Mosaic images from rapeseed and wheat breeding experiments were used for classification, and 10 DCNNs for lodging detection were trained using the transfer learning method. The proposed model significantly reduced the number of parameters while ensuring prediction, which makes the proposed model suitable for applications such as fast real-time classification while reducing the hardware requirements for high-throughput crop phenotyping.

Affected by factors such as field management, weather conditions and crop diseases, lodging often occurs in different growth stages of wheat, with the lodging characteristics varying among different stages. The currently available methods of extracting crop lodging plots are mainly based on data about a single growth stage. Without considering the different lodging characteristics of various crop growth stages, it is hard to apply this method to the actual monitoring of agricultural production. To solve these problems, it is particularly important to design a model that can monitor the lodging information of crops at various growth stages. In this study, we addressed the following two questions: (1) calculating the wheat lodging area and (2) analyzing wheat lodging areas at various growth stages. Additionally, the Transformer is a classical model originally used in natural language processing to improve the efficiency of machine translation. It has gradually been applied to the field of computer vision, including image segmentation. In this study, we applied the SegFormer model based on the Transformer to monitor wheat lodging for its high efficiency, great performance, strong robustness and lightweight [25].

## 2. Materials and Methods

### 2.1. Construction of Datasets

#### 2.1.1. Data Acquisition

**Figure A1:**
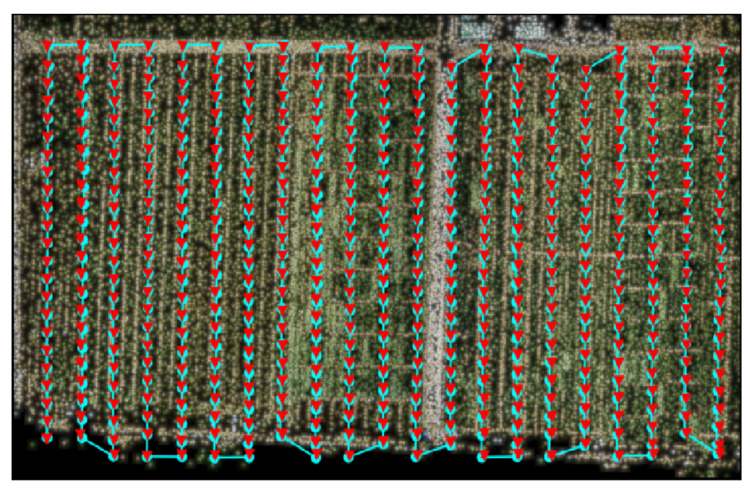
The flight position and angle of the UAV. Considering the UAV’s battery life and the plot area, the flying speed was set as 2.4 m/s, the course overlap rate was set as 80%, the side overlap rate was set as 75%, the shooting interval was set as 2 s, the ground resolution of UAV images was 0.7 cm/pixel, and the flight height of the UAV was set as 25 m.

The data used in this study were collected from the Agricultural Experimental Station of Shandong Agricultural University in Tai’an City, Shandong Province, China. Located at 117°9′14′′ N, 36°9′39′′ E, this area has a temperate monsoon climate with annual precipitation of 680 mm and an average annual temperature of 12.8 °C, which is favorable for the planting and production of wheat. The images were collected using a DJI Phantom 4 Pro V2.0 UAV, equipped with RGB sensors produced by Shenzhen DJ Innovation Industry Co., Ltd. (Shenzhen, China) The UAV’s flight route was planned using the two-dimensional map synthesis module of DJI GS Pro software. Figure A1 in Appendix A shows the flight position and angle of the UAV in a single shot.

The data were collected from different growth stages of wheat. From late May to early June, strong convective weather, such as thunder, lightning and strong wind, occurred many times in the Taishan District of Tai’an City, with the maximum wind speed reaching the scale of a fresh gale. In addition, severe weather, such as hail, resulted in many wheat lodging disasters at the experimental station. From 19 May 2021, to 9 June 2021, the UAV was used to constantly monitor wheat lodging, and the data were collected 2 to 3 times a week. Altogether, 8 groups of wheat lodging images were acquired, and relevant meteorological data were recorded. Table 1 shows the data acquisition information recorded after the occurrence of wheat lodging.

**Figure 1:**
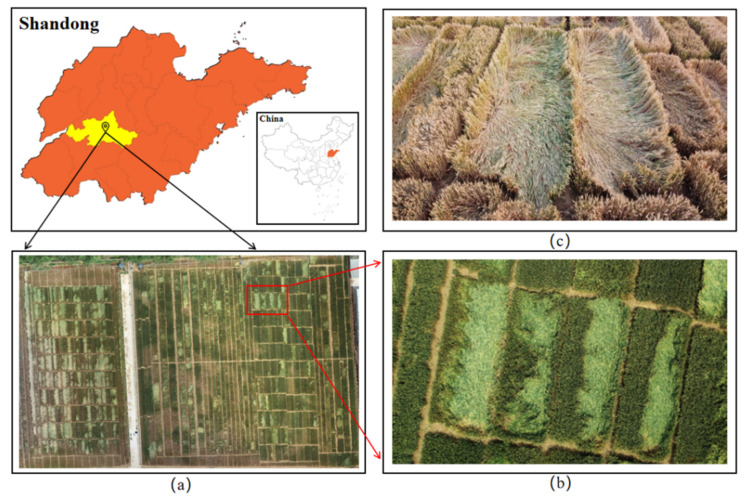
Wheat lodging images. (**a**) The study area located at the Agricultural Experimental Station of Shandong Agricultural University. (**b**) Partially enlarged details of wheat lodging images shot from the UAV. (**c**) Wheat lodging images shot from the ground.

Figure 1 shows the location of the study area, partially enlarged details of wheat lodging images shot from the UAV and wheat lodging images shot from the ground.

**Figure 2:**
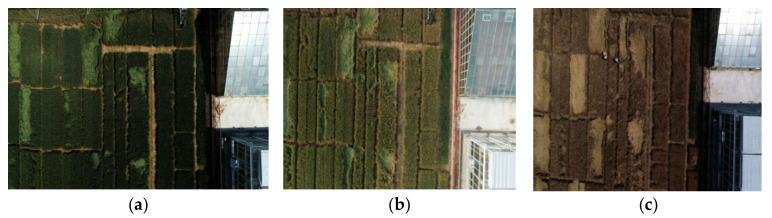
Lodging images of the same plot in different periods. (**a**) In lodging period I, the ears and leaves of wheat are generally green. (**b**) In lodging period II, the color of the wheat gradually turns from green to yellow from the tip of the wheat to its leaves, with part of the leaves remaining green. (**c**) In lodging period III, the wheat turns yellow from top to bottom, which is a sign of harvest.

In total, 3469 original images of the wheat field and the surrounding area were obtained. These images were classified into three categories according to three growth periods of wheat: milk period (lodging period I), dough period (lodging period II) and ripening period (lodging period III). Table 2 shows the statistical table of lodging data in each period, including 1220 images in lodging period I, 1397 images in lodging period II and 852 images in lodging period III. Figure 2 shows the lodging images of the same plot in different periods.

#### 2.1.2. Data Preprocessing

Before training and testing the image data in the model, to remove irrelevant information in the images, enhance the feature information correlated with lodging and simplify the data to the greatest extent, a multitude of preprocessing operations, including image mosaic, data screening and cutting, dataset annotation and data enhancement, were conducted on the image data.

(1) Image mosaic

**Figure A2:**
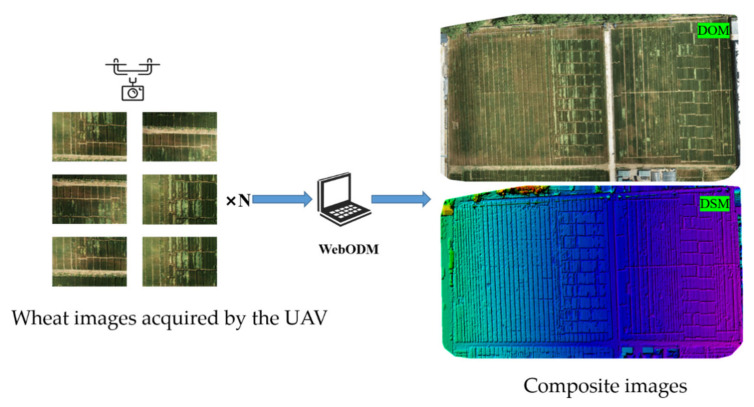
WebODM image mosaic process. WebODM automatically identified the coordinate information of each lodging image to eventually obtain the ground resolution of the DOM and DSM of the whole study area, i.e., 0.7 cm/pixel.

Several images of wheat fields collected by the UAV with a certain overlapping rate were mosaiced by feature matching. Restricted by the resolution of airborne sensors, the UAV’s flight height should be lowered to obtain more ground features. In this study, a single image taken by the UAV flying at a low altitude failed to cover the whole study area. WebODM, the fully automatic, fast and high-precision UAV data processing software, was used to mosaic the RGB images of the wheat collected by the UAV to finally obtain a digital orthographic image (DOM) and a digital surface model (DSM) covering the whole study area. Figure A2 shows the WebODM image mosaic process. In this study, wheat lodging in the whole plot was monitored based on the DOM of wheat lodging.

(2) Data screening and cutting

To improve the training effect of the deep learning model, the images were screened to eliminate unqualified ones. For example, images without wheat lodging and images with distortion and local overexposure caused by interference were removed, thereby enhancing the quality of the data inputted into the model.

The resolution ratio of a single original wheat lodging image acquired by the UAV is 5472 × 3648 but is limited by hardware conditions. Images of such a resolution ratio may exhaust the computer’s GPU resources during the training process. To prevent the feature information and spatial resolution of wheat lodging images from being affected by the limitation of hardware conditions, each wheat lodging image was cut into four 2736 × 1824 segments. Moreover, the details of the image to be annotated were enlarged so that the edge of the image could be observed and processed more effectively, thus improving the accuracy of the subsequent image annotation.

(3) Dataset Annotation

The interactive segmentation annotation tool of the open-source program EISeg [26] was applied to manually annotate the wheat lodging data. Efficient interactive segmentation (EISeg) refers to an efficient and intelligent interactive segmentation and annotation computer program developed based on PaddlePaddle. With high precision and lightweight interactive segmentation models applicable to different scenes, EISeg can achieve faster, more accurate and lower-cost annotation compared with the other popular annotation methods of semantic segmentation datasets. In addition, EISeg can apply the obtained annotated files to the semantic segmentation model provided by PaddleSeg [27] for training to gain a high-precision model suitable for customized scenes, realizing the whole process of segmentation tasks from data annotation to model training and prediction.

**Figure 3:**
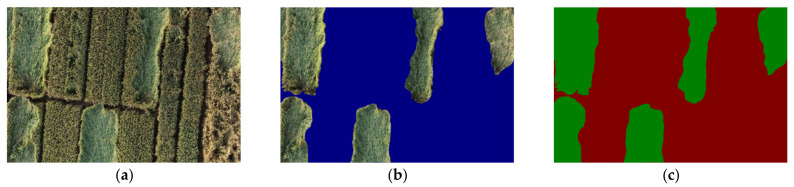
Dataset annotation results. (**a**) Original image. (**b**) Image obtained after image matting. (**c**) Pseudo-color image. The color palette was injected based on the original single-channel grayscale image to display the color effect without increasing the size of the image.

Figure 3 shows the dataset annotation results.

(4) Data enhancement

**Figure 4:**
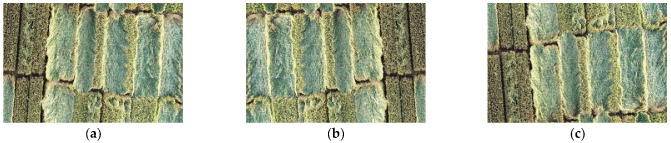

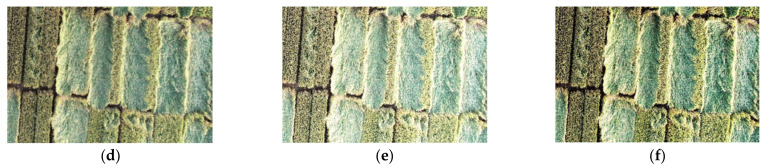
The dataset expanded by image enhancement. (**a**) The original image. (**b**) Horizontally flipped image. (**c**) Vertically flipped image. (**d**) A Gaussian filter was added to the image for fuzzy processing to strengthen the model’s ability to generalize fuzzy images. (**e**) Different levels of brightness conversion were evaluated to prevent the model from being affected by the diversity of light in the field. (**f**) The contrast of the wheat lodging image was enhanced to better express the texture detail at the edge of the image.

Insufficient data can lead to overfitting when the neural network is used for training. Therefore, it is necessary to perform data amplification operations, including (1) converting brightness at different levels to simulate the diversity of field ambient light; (2) improving the contrast of the image to show better texture details at the edge of the wheat lodging image; (3) filtering the images with Gaussian filter to enhance the generalization ability of the model to the blurred image; and (4) flipping the image horizontally and vertically to expand the number of images. Figure 4 shows the original image and the expanded wheat lodging images. After the expansion, the total number of wheat lodging images reached 8000.

### 2.2. Extracting Wheat Lodging Plots

#### 2.2.1. Training, Validation and Testing Dataset

A total of 7200 images were randomly selected from the 8000 images to form the training and validation datasets of the model. The training dataset contained 6400 images, the validation dataset consisted of 800 images and the remaining 800 images served as the testing dataset.

#### 2.2.2. Establishment of the SegFormer Semantic Segmentation Model

In wheat lodging detection, in addition to manual monitoring, machine learning methods rely on information such as the shape and color of lodging plots to extract complicated features of lodging wheat. Moreover, the irregular shapes of lodging plots make it even more complicated to extract these features. Currently, popular CNN-based semantic segmentation models cannot guarantee the segmentation effect while reducing the number of network parameters.

**Figure 5:**
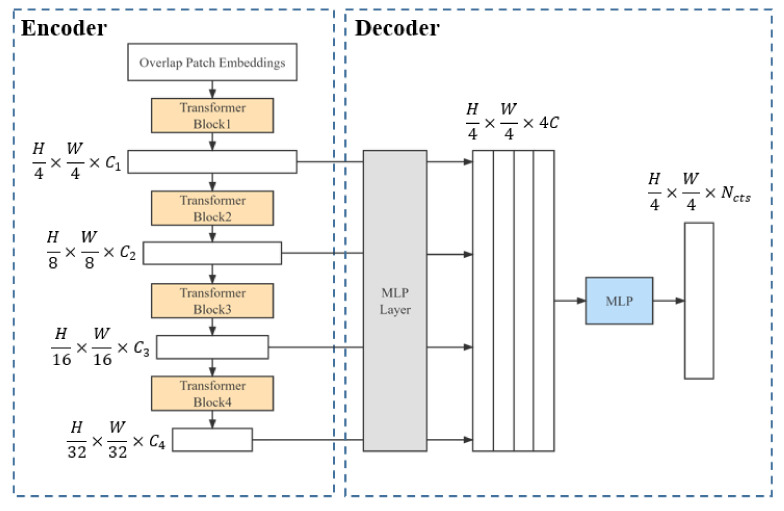
SegFormer model structure. The SegFormer model consists of an encoder and a decoder. The encoder mainly includes a multi-layered Transformer module for generating high-resolution coarse features and low-resolution fine features, and the decoder includes a lightweight multi-layered perceptual decoder for fusing multi-level features to generate the final mask of semantic segmentation.

The SegFormer model is a simple and efficient semantic segmentation method based on the Transformer network. Compared with the current mainstream semantic segmentation models, the SegFormer model is efficient, high-performance and lightweight [25]. Figure 5 shows the structure of the SegFormer model. A given image is divided into several smaller segments and inputted into the multi-layered Transformer encoder to obtain the multi-layered features of the original image and then transfer these features to the lightweight multi-layered perceptual decoder to generate the final mask.

In deep learning, the optimizer minimizes the loss function by training the optimization parameters. The loss function was used to calculate the deviation between the real value and the predicted value of the target value in the test set. As an algorithm improved based on the regularization of Adam + L2, AdamW has effectively strengthened the generalization performance of the Adam optimizer. Therefore, in this study, AdamW was used as the optimizer of the SegFormer model to update its network parameters. Polynomial decay, the interpolated value between the initial learning rate and the final learning rate, was set as the learning rate, which was calculated based on the weight of the polynomial. During the training process, the loss function can measure the performance of the model and obtain the difference between the model’s estimated results and the actual data. In addition, the Cross-Entropy Loss function was used as the criterion to measure the segmentation effect of the SegFormer model, and the SegFormer-B0 and SegFormer-B1 models with the smallest number of parameters were selected.

#### 2.2.3. Establishment of the DeepLabv3+ Semantic Segmentation Model

To further explore the performance of the SegFormer model in wheat lodging datasets, a comparative experiment was carried out in this study.

Combining the strengths of the ASPP module and the encoder–decoder structure, the DeepLabv3+ model can improve the segmentation effect of the target boundary to gain clearer boundaries of the segmented objects on the premise of effectively capturing more metric information. Hence, in this study, the DeepLabv3+ model was applied for comparison.

**Figure 6:**
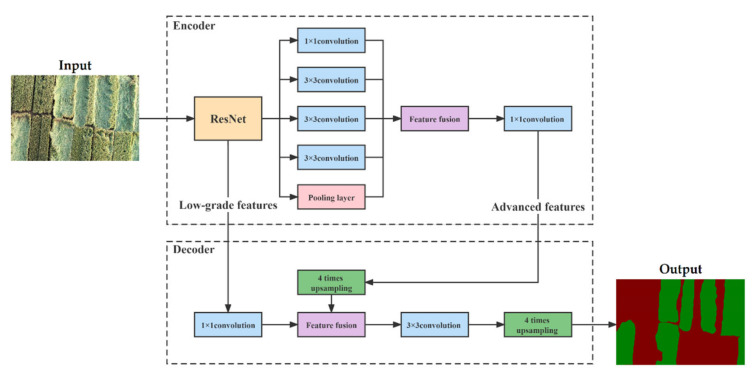
DeepLabv3+ model structure. The ASPP structure consists of one 1 × 1 convolution and three 3 × 3 convolutions, all of which have 256 convolution kernels. After processing, the output feature map contains 256 channels.

The original DeepLabv3+ uses Xception as the DCNN to extract image features. To make the model less complex, ResNet50 and ResNet101, two backbone networks in ResNet, served as the backbone feature extraction networks of the DeepLabv3+ semantic segmentation model. Figure 6 shows the structure of the DeepLabV3+ model based on backbone networks in ResNet.

Stochastic gradient descent with momentum (Momentum-SGD) was used as the optimizer of the DeepalBV3+ model. Similar to the SegFormer model, polynomial decay was set as the learning rate of the DeepalBV3+ model, and the Cross-Entropy Loss function also served as the criterion to measure the model’s segmentation effect.

#### 2.2.4. Setting of the Two Models’ Training Parameters

To gain a better learning effect, the semantic segmentation network was trained by transfer learning. Table 3 shows the basic parameters set for the training of the two models.

### 2.3. Analyzing Wheat Lodging Monitoring at Various Growth Stages

#### 2.3.1. Training, Validation and Testing Dataset

**Figure A3:**
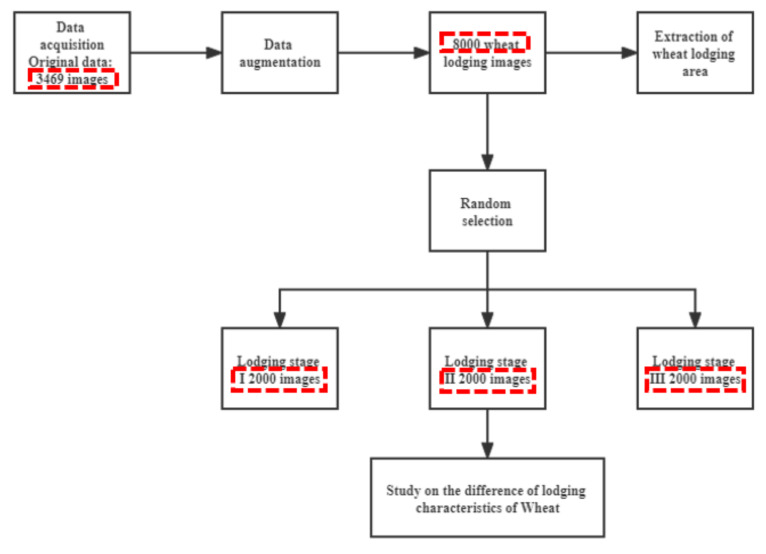
The schematic diagram of the data set construction process.

Based on 8000 images, 2000 images were randomly selected in each stage, and a total of 6000 wheat lodging images in stages I (2000), II (2000) and III (2000) were obtained (Figure A3). The training and validation datasets were formed by 1800 images randomly selected from each stage, with the training dataset containing 1600 images and the validation dataset containing 200 images. The remaining 200 images constituted the testing dataset. In addition, 1000 wheat lodging images were randomly selected from the dataset of each stage to establish a mixed dataset that included 3000 images altogether. The ratio of images in the training dataset, validation dataset and testing dataset is 8:1:1. Table A1 shows the statistical lodging data for each period.

#### 2.3.2. Selection of the Deep Learning Algorithm

To accurately evaluate wheat lodging plots in different periods, the semantic segmentation models used to extract wheat lodging plots in different periods were explored. In this study, multiple deep learning-based semantic segmentation algorithms were applied to compare the multi-temporal segmentation of wheat lodging plots. Eight common semantic segmentation models, SegFormer-B1, UNet, PSPNet, OCRNet, FCN, Fast-SCNN, DeepLabv3+ and DANet, were used to conduct comparative experiments in the datasets of different periods.

#### 2.3.3. Setting of Model Training Parameters

During the training process, the value of the target categories num_classes was set to be 2, with lodging wheat and the background being the targets. Table A2 shows the basic parameter settings of each model.

### 2.4. Evaluation Indices

(1) Accuracy (ACC): ACC is the most commonly used evaluation index in semantic segmentation. It refers to the ratio of the number of correctly predicted pixels to the total number of pixels. The higher the ACC, the more reliable the model. ACC can be calculated using Equation (1).

(1) $$\mathrm{ACC}=\frac{\mathrm{TP}+\mathrm{TN}}{\mathrm{TP}+\mathrm{TN}+\mathrm{FP}+\mathrm{FN}}$$

(2) Mean Intersection over Union (mIoU): The dataset of each category predicted by the model was calculated separately. mIoU is the mean of the intersection between the predicted area and the actual area divided by the union of the predicted area and the actual area of all categories, as shown in Equation (2). In this study, the maximum value of mIoU served as the criterion for saving the optimal model.

(2) $$\mathrm{mIoU}=\frac{\mathrm{TP}}{\mathrm{FP}+\mathrm{FN}+\mathrm{TP}}$$

(3) Kappa coefficient: Used in the consistency test, the Kappa coefficient usually ranges from 0 to 1. The coefficient can be divided into five groups to represent different levels of consistency: 0.0–0.20 indicates extremely low consistency, 0.21–0.40 indicates general consistency, 0.41–0.60 indicates medium consistency, 0.61–0.80 indicates high consistency and 0.81–1 indicates almost complete consistency. The Kappa coefficient can be calculated based on Equation (3), where p_0_ denotes the sum of correctly classified samples of each category divided by the total number of samples, i.e., the overall classification accuracy, and p_e_ is the sum of “the product of actual and predicted quantities” corresponding to all categories divided by “the square of the total number of samples”.

(3) $$\mathrm{Kappa}=\frac{p_{0}-p_{e}}{1-p_{e}}$$

(4) Dice coefficient: As a function that measures the similarity between different sets, the Dice coefficient is usually used to calculate the similarity between two samples. Ranging from 0 to 1, the Dice coefficient can be calculated using Equation (4).

(4) $$\mathrm{Dice}=\frac{2\times \mathrm{TP}}{\mathrm{FP}+2\times \mathrm{TP}+\mathrm{FN}}$$

where true positivity (TP) indicates that the prediction is correct, and both the predicted value and the truth value are wheat lodging; that is, the correctly detected wheat lodging plots. False positivity (FP) means that the prediction is wrong; that is, the predicted value is wheat lodging, but the truth value is the background. True negativity (TN) indicates that the prediction is correct, with both the predicted value and the truth value is the background. False negativity (FN) implies a prediction error; that is, the predicted value is the background, while the truth value is wheat lodging. In this study, the multi-scale rollover evaluation method and the sliding window evaluation method were used to assess the model. Multi-scale rollover evaluation refers to horizontally and vertically flipping the images before inputting the images and annotated data into the model with the purpose of expanding the validation dataset. In the sliding window evaluation, the window slides from left to right and from top to bottom at a given step size, and the current window area is predicted and evaluated every time the window slides.

## 3. Results and Analysis

### 3.1. Wheat Lodging Area Extraction

#### 3.1.1. Model Training

**Figure 7:**
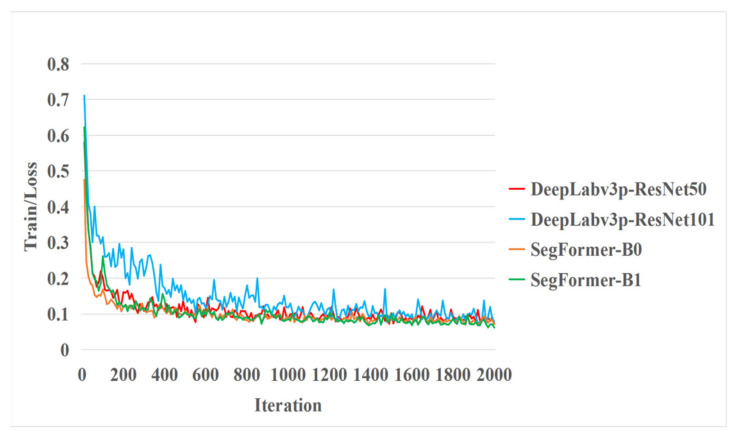
Visualization of the training process for each model.

During the training process (Figure 7), the model’s loss value gradually decreased as the number of iterations increased. At the initial stage of model training, the model’s learning rate is relatively large, so the loss curve converges comparatively quickly. However, with an increase in the number of iterations, the learning rate constantly declines, and the slope of the loss curve also gradually decreases. In the end, the fluctuation trend of the final loss value is gradually stabilized, the loss ratio no longer declines, and the model reaches convergence at this time.

With an initial learning rate of 0.001, the two SegFormer models have the same decline strategy; the initial learning rate of the two backbone network models of DEPLABV3+ is 0.01, so they share the same decline strategy. Hence, the two models corresponding to SEGFER and DEPLABV3+ have completely consistent learning rate curves.

Figure 7 shows that, given the same hardware resources, the SegFormer-B0 model, SegFormer-B1 model and DeepLabv3+ model based on the ResNet50 backbone network, with their loss curves declining more rapidly, converged within a shorter period of time during the training process.

#### 3.1.2. Model Evaluation

**Figure 8:**
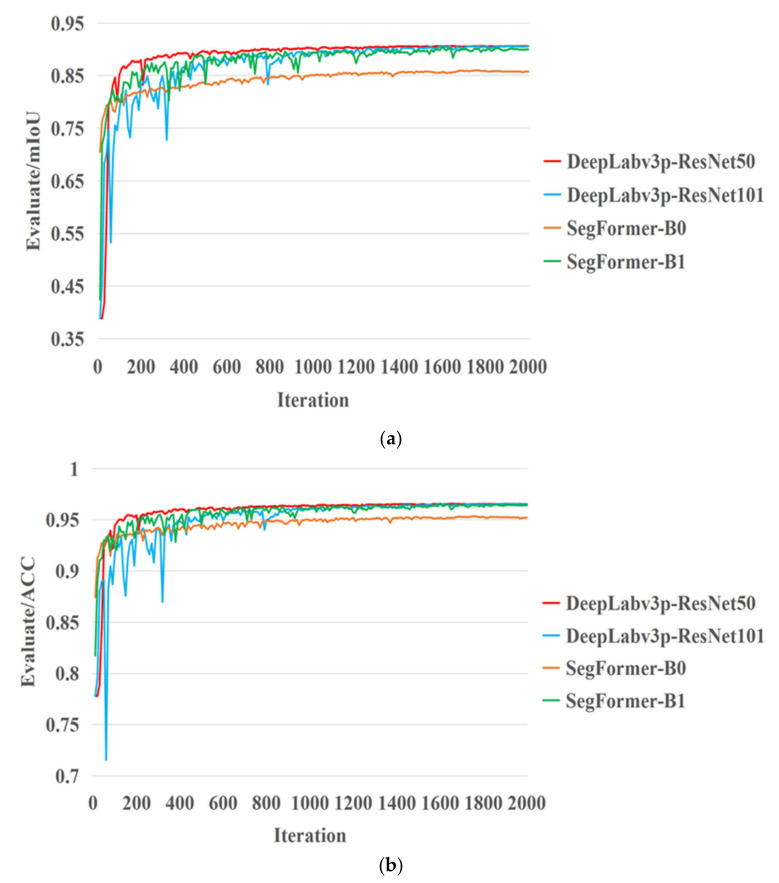
Model evaluation visualization. (**a**) Visualization of mIoU, with Evaluate/mIoU denoting the mIoU of the validation dataset during the training process. (**b**) Visualization of ACC, with Evaluate/ACC representing the accuracy of the validation dataset during the training process.

During the training process, the weight files generated in the training process were evaluated by the validation dataset every 10 iterations, and the indices Evaluate/mIoU and Evaluate/ACC obtained in the evaluation process were visualized. Figure 8 shows a visualization of the model evaluation.

As shown in Figure 8, the ACC and mIoU of the validation dataset constantly increased as the number of iterations increased. Although the evaluation indices fluctuated during period I of the training process, the overall curve still remained under an upward trend. As shown in Figure 8, in the wheat lodging dataset, the evaluation indices of the SegFormer-B1 model were significantly higher than those of the SegFormer-B0 model and quite similar to those of the DeepLabv3+ model. Compared with the ResNet101 backbone network, the DeepLabv3+ model with ResNet50 as the backbone network not only converged at a faster rate but also had an ACC and mIoU slightly higher than those of the DeepLabv3+ model with ResNet101 as the backbone network.

#### 3.1.3. Model Prediction

The optimal weight files generated by the SegFormer and DeepLabv3+ models were used to evaluate and predict the wheat lodging dataset. 

Table 4 shows the performance of the SegFormer-B1 model in the training validation dataset and the testing dataset. ACC reached 96.56%, suggesting that the model achieved satisfactory performance. 

**Figure 9:**
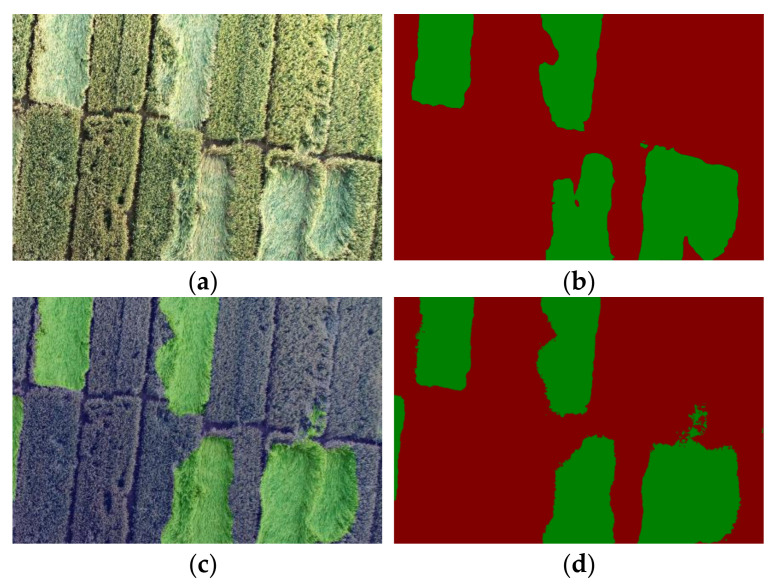
Model prediction. (**a**) Original image. (**b**) Manually annotated image. (**c**) Image predicted by the model. (**d**) Predicted annotated image. The image predicted by the model is a mixture of the original image and the predicted annotated image.

In the prediction process, the weight file with the highest mIoU obtained by the SegFormer-B1 model in the validation dataset was selected as the optimal weight, and the wheat lodging images in the testing dataset were predicted as the optimal weight of the model. Figure 9 shows the model’s segmentation results in the wheat lodging testing dataset, including the original image, the manually annotated image, the image predicted by the model and the predicted annotated image. Since the images were segmented based on the image features extracted in the training process, the model adhered to a more uniform standard to evaluate all images, which overcame the strong subjectivity in manually judging the wheat lodging plots.

#### 3.1.4. Wheat Lodging Area Analysis

**Figure 10:**
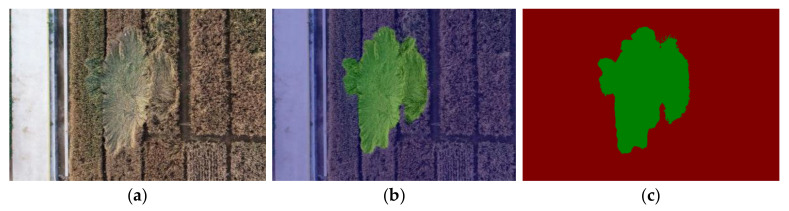
Extraction of the wheat lodging area. (**a**) Original wheat lodging image. (**b**) Image predicted by the model. (**c**) Annotated image predicted by the model. The annotated image predicted by the model has the same resolution as the original image input into the model.

Wheat lodging can severely reduce wheat yield; thus, it is of great significance to evaluate wheat lodging plots. As shown in Figure 10, the annotated image predicted by the SegFormer-B1 model is a pseudo-color image only in two colors, namely green (the lodging plots) and red (the non-lodging plots). In addition, the pseudo-color image has the same resolution as the original image inputted into the model. Therefore, the annotated image predicted by the model was quite suitable for calculating the wheat lodging area. The annotated image predicted by the model had a resolution of 3022 × 1906. In the process of image data collection, the flying height of the UAV was 25 m, and the corresponding ground resolution was 0.7 cm/px; that is, a single pixel in the image corresponding to a 0.7 cm × 0.7 cm square on the ground, which means that a single pixel in the image is equivalent to an actual ground area of 0.49 cm^2^.

The NumPy numerical calculation library and the OpenCV image processing library in Python were applied to calculate the proportion of different colors and the number of pixels in the annotated image predicted by the model. Table 5 shows the statistics for the wheat lodging area. As shown in Table 5, in the annotated image predicted by the model, there were 924,477 green pixels (lodging plots), accounting for 16.05%, and 4,835,455 red pixels (non-lodging plots), accounting for 83.95%. This indicates that the actual wheat lodging area in this predicted image is 924,477 × 0.49 cm^2^, which is about 45.29 m^2^.

#### 3.1.5. Model Evaluation with Additional Dataset

**Figure A4:**
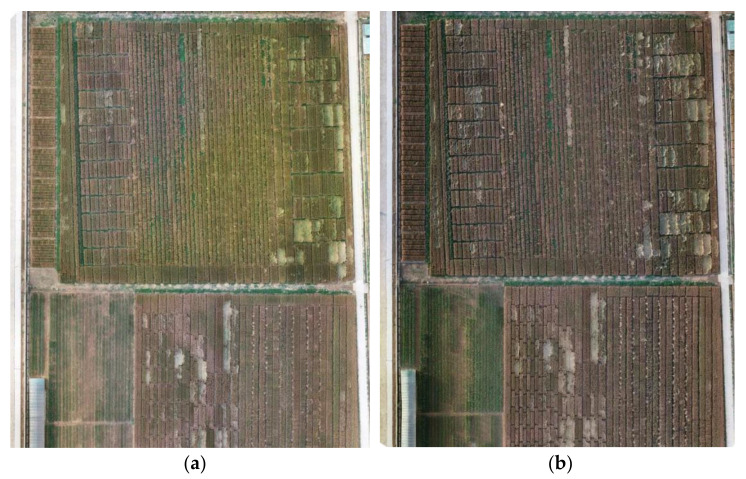
Images of adjacent parcels. (**a**) milk period I. (**b**) ripening period III. To verify the model’s generalization performance, lodging wheat images of adjacent parcels in different periods were collected by the UAV.

To further verify the generalization performance of the model, in addition to establishing the test dataset, in the process of data collection, this study used drones to collect multiple periods of adjacent plots (117°9′59′′ N, 36°9′55′′ E) of the research site in the test station. The lodging wheat image data aimed to provide enough fresh samples for the model to verify the generalization performance of the model. Image stitching was performed on the image of the plot, and a multi-period orthophoto image of the plot was obtained, as shown in Figure A4.

**Figure A5:**
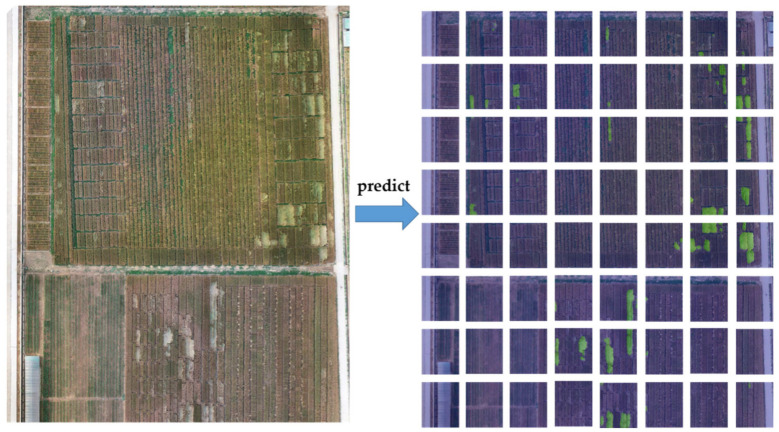
Model predictions. The resolution of mosaic images, which are often large, may cause problems, such as memory overflow in the prediction, which puts forward strict requirements on hardware resources. Hence, cutting the generated RGB DOM into several small segments can greatly reduce the requirements of the model for hardware resources.

The preprocessed images of adjacent parcels were inputted into the model for evaluation and prediction. Table 6 shows the evaluation results, and Figure A5 shows the model predictions. This model achieved an ACC and mIoU of 96.52% and 89.66%, respectively, in the adjacent parcels, which implies that the model has an ideal segmentation effect among the fresh samples, indicating a strong generalization performance of the model.

### 3.2. Wheat Lodging Analysis in Various Growth Stages

#### 3.2.1. Model Training

After the training, the testing datasets of three different periods were used to test the models of different periods, and the evaluation results are shown in Table 7. In the process of data labeling, the wheat lodging images are divided into two categories: Background and Lodging. The ACC index in Table 7 is the accuracy of the Lodging category in the wheat lodging image.

As shown in Table 7, the DANet model had the best performance in the testing dataset in lodging period I, with its mIoU reaching 88.91%. In the testing dataset in lodging period II, the model with the optimal performance was also DANet, with its mIoU reaching 90.56%. In terms of the testing dataset based on lodging period III, the DANet and PSPNet models had the best performance. The mIoU of the DANet model reached 89.92%, and the PSPNet model had a mIoU of 89.70%. The results predicted by the two models were quite similar. Among the testing datasets for all three periods, the lightweight Fast-SCNN real-time semantic segmentation model, with a simplified structure and decreased parameters, performs poorly in every wheat lodging period.

After the model was tested using the test datasets of three different lodging periods, the model built on the mixed dataset containing the three periods was tested, and the results are shown in Table 8. ACC evaluation metrics in Table 8 were calculated based on overall accuracy.

Table 8 shows that the five models achieve an ACC of greater than 96% in the mixed dataset, with DANet model having the best performance as evaluated by various indices, achieving the highest ACC of 97.14%.

#### 3.2.2. Influences of Different Lodging Periods on the Model Segmentation Effect

**Figure A6:**
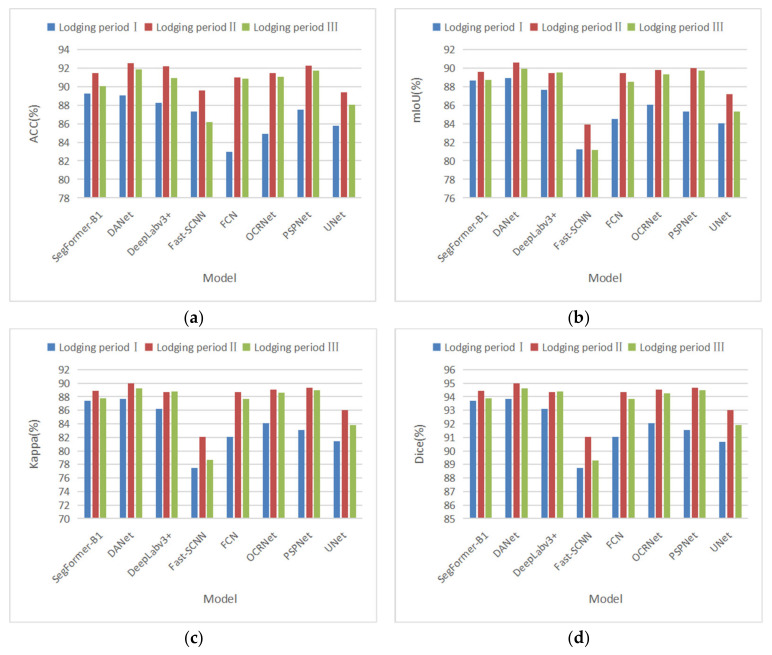
Horizontal comparison of the segmentation effect of different models in datasets of different lodging periods. The models were tested in the datasets of periods I, II and III to horizontally compare their accuracy. (**a**) Comparison results of accuracy of each model in the lodging datasets in different periods. (**b**) Comparison results of mIoU of each model in the lodging datasets in different periods. (**c**) Comparison results of Kappa of each model in the lodging datasets in different periods. (**d**) Comparison results of Dice of each model in the lodging datasets in different periods.

Figure A6 shows a horizontal comparison of the segmentation effects of different models in datasets of different lodging periods. As shown in Table 7 and Figure A6, in most cases, the models performed the best in lodging stage II, followed by lodging stage III, and the worst in lodging stage I. Better extraction of lodging features. In the actual production process, there may be many factors that affect the segmentation effect of the model. The color of wheat will gradually change from green to yellow-green and finally to yellow with continuous growth throughout its growth cycle. During this process, the color of wheat lodging between the area and the non-lodging area will also change, however, as wheat grows, the factors that induce wheat lodging are also increasing. In this process, in addition to the increasing lodging area, the lodging direction of wheat may also change. For example, the wind direction changes when lodging occurs, and the texture of the wheat lodging area becomes more complex, which may also affect the model segmentation effect.

#### 3.2.3. Model Comparison for Combined Datasets from Different Stages

**Figure 11:**
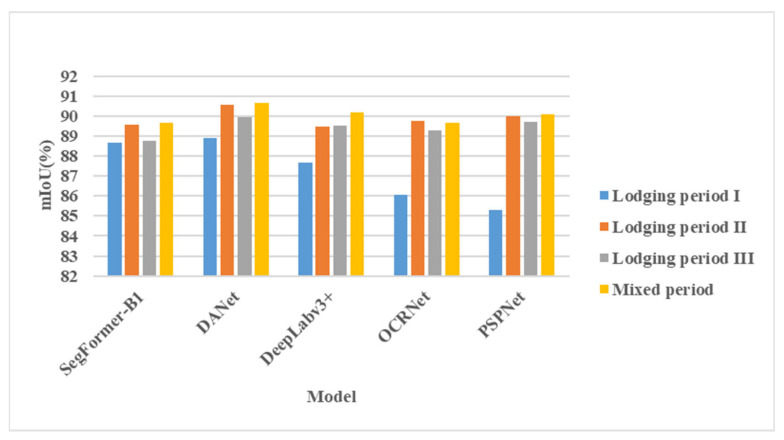
Comparison of model mIoU evaluation indices based on multi-source datasets. A comparison of different models based on single-period datasets and mixed datasets in terms of mIoU is presented.

Figure 11 shows a comparison of different models in terms of mIoU, based on single-period datasets and mixed datasets of different periods. In most cases, the performance effect of the multi-period mixed dataset model had the smallest difference with the evaluation indicators of the lodging stage II model, followed by the lodging stage III model. The mIoU of the lodging stage I model had the largest difference. In the PSPNet model, the difference reached 4.79%. The model with the smallest difference was SegFormer-B1, and the difference in mIoU was 0.97%, indicating that compared with other models, the SegFormer-B1 model had a better effect on the characteristics of lodging wheat in the datasets of each period. As shown in Figure 11, in most cases, the model based on the mixed dataset not only accurately extracted the wheat lodging area at each stage of wheat growth but also obtained a higher evaluation index than the single-period lodging dataset, which may be because the model extracted more wheat lodging features in the mixed-period dataset compared to the single-period dataset; therefore, the mIoU indicator of the model also increased accordingly. In summary, the SegFormer-B1 model obtained based on the mixed dataset can be applied to the extraction of wheat lodging plots in all growth stages of wheat, holds great potential for application in actual agricultural production and can serve as a powerful tool for monitoring wheat lodging.

## 4. Discussion

### 4.1. Extraction Method of Wheat Lodging Area Based on Deep Learning

The SegFormer model based on the MixVIT backbone network and the DeepLabv3+ model based on the ResNet backbone network were constructed using the transfer learning method. The prediction speeds of the two models were compared with various evaluation indicators in the wheat lodging dataset. It is suitable for the actual production process of real-time monitoring of wheat lodging. The SegFormer-B1 model, based on the MixVIT backbone network, was used to train and test the wheat lodging dataset. The detection accuracy of the wheat lodging dataset reached 96.56%. The label file of the lodging wheat obtained by the model segmentation was used for lodging. The calculation of the wheat area achieved an accurate measurement of the wheat lodging area.

### 4.2. Difference in Characteristics of Multi-Temporal Wheat Lodging Datasets

Based on the multi-temporal wheat lodging dataset collected by UAV, the dataset was divided into three categories according to the different growth stages of wheat when lodging occurred, and eight semantic segmentation models were used to train and test the lodging datasets of the three periods. To explore the effectiveness of the single-period dataset, a mixed-period wheat lodging dataset was established and inputted into the semantic segmentation model for training and testing. The experimental results showed that with the development of the lodging period, the segmentation effect of the model mostly increased first. After the downward trend, it performed best in the lodging phase II dataset. Generally speaking, the segmentation effect of the model based on the single-period dataset was lower than that of the model based on the mixed dataset. Finally, the comparison of the models trained in the mixed dataset showed that the SegFormer-B1 model can be a good balance between the segmentation accuracy of the model.

## 5. Conclusions

In this study, a wheat lodging monitoring method applicable to the whole growth cycle of wheat was proposed based on deep learning. This method was verified to be effective in segmentation, fast in prediction and strong and robust in generalization. In addition, using a UAV to collect wheat lodging images and then monitoring wheat lodging and calculating lodging areas based on image information can not only realize non-destructive monitoring of wheat lodging but also is applicable to scenarios with high requirements for real-time performance and accuracy of disaster monitoring, e.g., crop loss and damage response and subsequent settlement of agricultural insurance claims.

In the next step, to make the work available for agricultural insurance, further efforts will be made to construct an online wheat lodging plot extraction system, which will be combined with UAV flight and image mosaic technologies to provide an effective tool for monitoring wheat lodging in large areas.

## Figures and Tables

**Table 1:** Data acquisition information record.

Date	Time	Weather	Temperature (°C)	Wind Direction/Strength
19 May	10:00–14:00	cloudy	14~25	gentle southerly breeze
22 May	10:00–14:00	cloudy	20~30	gentle southerly breeze
25 May	10:00–14:00	cloudy	15~28	moderate southwesterly breeze
27 May	10:00–14:00	sunny	17~31	gentle southwesterly breeze
28 May	10:00–14:00	sunny	18~28	gentle northwesterly breeze
31 May	10:00–14:00	cloudy	20~29	light northeasterly breeze
3 June	10:00–14:00	cloudy	14~24	light northwesterly breeze
9 June	10:00–14:00	sunny	22~36	gentle southerly breeze

**Table 2:** Statistical table of lodging images in each period.

Wheat Growth Stage	Data Size (Number of Images)	Lodging Period
milk	507	lodging period I
milk	214	lodging period I
milk	499	lodging period I
dough	505	lodging period II
dough	507	lodging period II
dough	385	lodging period II
ripening	348	lodging period III
ripening	504	lodging period III

**Table 3:** Model training parameter settings.

Model	Iteration	Batch_Size	Optimizer	Learning_Rate	Loss
SegFormer	2000	16	AdamW	0.001	CrossEntropyLoss
DeepLabv3+	2000	8	Momentum-SGD	0.01	CrossEntropyLoss

**Table 4:** Evaluation of the model performance effect.

SegFormer-B1	Training Set	Test Set
ACC (%)	96.76	**96.56**
mIoU (%)	89.99	89.57
Kappa (%)	88.62	88.38
Dice (%)	94.31	94.19

**Table 5:** Statistics of wheat lodging area.

Label Color	Number of Pixels	Occupied Area	Proportion
Green	**924,477**	45.29 m^2^	**16.05%**
Red	**4,835,455**	236.94 m^2^	**83.95%**

**Table 6:** Evaluation of the performance effect of the adjacent plot model.

SegFormer-B1	Training Set	Test Set (Adjacent Parcels)
ACC (%)	96.76	**96.52**
mIoU (%)	89.99	**89.66**
Kappa (%)	88.62	88.45
Dice (%)	94.31	94.23

**Table 7:** Evaluation results of the models in each period.

Model	Lodging Period	ACC (%)	mIoU (%)	Kappa (%)	Dice (%)
		Test Set			
SegFormer	I	89.25	88.67	87.43	93.71
	II	91.46	89.56	88.85	94.42
	III	90.08	88.75	87.78	93.89
DANet	I	89.05	88.91	87.70	93.85
	II	92.53	90.56	89.96	94.98
	III	91.86	89.92	89.24	94.62
DeepLabV3+	I	88.24	87.68	86.18	93.09
	II	92.18	89.47	88.71	94.35
	III	90.90	89.51	88.77	94.39
Fast-SCNN	I	87.29	81.23	77.50	88.75
	II	89.60	83.92	82.10	91.03
	III	86.20	81.16	78.65	89.30
FCN	I	82.98	84.51	82.05	91.02
	II	90.97	89.45	88.70	94.35
	III	90.84	88.53	87.65	93.83
OCRNet	I	84.89	86.07	84.11	92.05
	II	91.46	89.76	89.06	94.53
	III	91.07	89.30	88.55	94.27
PSPNet	I	87.54	85.28	83.07	91.53
	II	92.28	89.99	89.31	94.65
	III	91.71	89.70	88.98	94.49
UNet	I	85.77	84.02	81.39	90.69
	II	89.37	87.16	86.03	93.01
	III	88.05	85.32	83.79	91.89

**Table 8:** Model performance evaluation based on mixed-period datasets.

Model	ACC (%)	mIoU (%)	Kappa (%)	Dice (%)
	Test Set			
SegFormer-B1	96.43	**89.64**	88.53	94.29
DANet	**97.14**	90.65	89.99	94.99
DeepLabv3+	97.00	90.16	89.42	94.71
OCRNet	96.82	89.65	88.83	94.42
PSPNet	96.98	90.07	89.32	94.66

## Data Availability

The data presented in this study are available on request from the corresponding authors.

## Appendix A

**Table A1:** Statistical table of lodging data at each stage.

Lodging Stage	Data Size (Number of Images)	Training Dataset (Number of Images)	Validation Dataset (Number of Images)	Testing Dataset (Number of Images)
Lodging stage I	2000	1600	200	200
Lodging stage II	2000	1600	200	200
Lodging stage III	2000	1600	200	200

**Table A2:** Training process model parameter setting.

Model	Iteration	Batch_Size	Optimizer	Learning_Rate	Loss
SegFormer-B1	2000	16	AdamW	0.001	CrossEntropyLoss
DANet	2000	8	Momentum-SGD	0.005	CrossEntropyLoss
DeepLabv3+	2000	8	Momentum-SGD	0.01	CrossEntropyLoss
Fast-SCNN	2000	32	Momentum-SGD	0.01	CrossEntropyLoss
FCN	2000	8	Momentum-SGD	0.005	CrossEntropyLoss
OCRNet	2000	8	Momentum-SGD	0.01	CrossEntropyLoss
PSPNet	2000	8	Momentum-SGD	0.01	CrossEntropyLoss
UNet	2000	8	Momentum-SGD	0.01	CrossEntropyLoss
